# Fabrication and Characterization of Graphene–Mesoporous Carbon–Nickel–Poly(Vinyl Alcohol)-Coated Mandrel-Coiled TCP_FL_^NR^ Artificial Muscle

**DOI:** 10.3390/biomimetics9080458

**Published:** 2024-07-26

**Authors:** Pawandeep Singh Matharu, Yuyang Song, Umesh Gandhi, Yonas Tadesse

**Affiliations:** 1 Humanoid, Biorobotics and Smart Systems (HBS Lab), Mechanical Engineering Department, The University of Texas at Dallas, Richardson, TX 75080, USA; yonas.tadesse@utdallas.edu; 2 Toyota Research Institute of North America, 1555 Woodridge Ave, Ann Arbor, MI 48105, USA; yuyang.song@toyota.com (Y.S.); umesh.gandhi@toyota.com (U.G.)

**Keywords:** soft actuator, twisted and coiled polymer muscles (TCP), coating, nanomaterial, smart material

## Abstract

This study investigates the performance enhancement of mandrel-coiled twisted and coiled polymer fibers with a nichrome heater (TCP_FL_^NR^) by coating with a solution of graphene–mesoporous carbon–nickel–polyvinyl alcohol. The coating process involved a one-pot synthesis utilizing graphene powder, Ni nanoparticles, mesoporous carbon, and PVA as a binding agent. The coating was performed by manually shaking the TCP_FL_^NR^ and the subsequent annealing process, which results in improved thermal conductivity and actuation behavior of the TCP_FL_^NR^. Experimental results on a 60 mm long actuator demonstrated significant enhancements in actuation displacement and actuation strain (20% to 42%) under various loads with an input current of 0.27 A/power 2.16 W. The blocked stress is ~10 MPa under this 2.16 W power input and the maximum strain is 48% at optimum load of 1.4 MPa. The observed actuation strain correlated directly with the input power. The coated TCP_FL_^NR^ exhibited better thermal contacts, facilitating enhanced heat transfer, and reducing power consumption by 6% to 9% compared to non-coated actuators. It was found that the nanomaterial coating helps the TCP actuator to be reliable for more than 75,000 actuation cycles at 0.1 Hz in air due to improved thermal conductivity. These findings highlight the potential for further research to optimize electrothermally operated TCP actuators and unlock advancements in this field.

## 1. Introduction

Soft artificial muscles that function similarly to natural muscles are crucial components for advanced applications such as soft robots (both in air and underwater), orthotics, prosthetics, humanoid robots, wearables, medical devices, and more. This is because traditional actuators like motors and pumps are bulky, noisy, and rigid [1,2]. Therefore, significant effort and research has been devoted to developing muscle-like actuators that are efficient, and possess high strain capabilities, high load-carrying capacity, dynamic mechanical compliance, and high specific energy/power. Many types of actuators have been proposed, including shape memory alloys [3,4,5], Cavatappi [6,7] (which are pneumatically or hydraulically driven and shaped like TCP), pneumatic actuators [8,9], electromagnetic actuators [10], thermally actuated twisted and coiled actuators (TCPs) [11,12,13,14,15,16], and others. The performance of most of these actuators depends on the input driver.

Haines et al. [11] developed a promising TCP actuators from fishing line capable of lifting objects 100 times heavier than a human muscle, with a contractile strain of 49%. They also demonstrated an efficiency of approximately 1% by wrapping CNT sheets over polyethylene fiber. The fundamental mechanism of a CNT actuator, displaying torsional and tensile actuation, was thoroughly investigated by Lee et al. [17] and Di et al. [18]. As noted by Higueras-Ruiz et al. [6], it was experimentally demonstrated that thermally driven actuators are less efficient and consume high power due to the low thermal conductivity of polymer fibers. TCPs operate based on Joule heating when an electric current is applied to them. Since polymer fibers have low thermal conductivity, CNTs have been utilized to fabricate coiled artificial muscles, which provide significant forces with rapid tensile actuation but pose challenges in fabrication due to technical complexities and high manufacturing costs [19]. Piao et al. [20] introduced nanomaterial coating with graphene flakes on silver-coated nylon fibers (nylon 6,6 sewing threads, 260151023534, Shieldex) to enhance dynamic performance and leverage inexpensive polymer fibers. Subsequently, to improve the cycle performance of these actuators, Piao and Suk [20,21] spray-coated the TCP_Ag_ polymer fibers with a graphene/silver nanoflower hybrid solution. They demonstrated a 38% reduction in total actuation cycle time and a threefold larger peak-to-peak amplitude of the displacement oscillation compared to non-coated TCP_Ag_.

Graphene’s unique 2D geometry gives rise to unusual thermal properties, paving the way for new discoveries in heat-flow physics and aiding in novel thermal management applications. According to Pop et al. [22] and Su et al. [23], the thermal conductivity of graphene, with its perfect structure, rivals that of diamond materials (>5000 W/mK). Additionally, graphene possesses a super-high aspect ratio and a 2D structural morphology, making it an ideal material as a filler for polymers to achieve very high thermal conductivity [24,25,26]. Monolayer graphene with minimal defects exhibits a remarkably high thermal conductivity of 2000 W/mK [27,28]. Therefore, to enhance the thermal conductivity of TCP_FL_^NR^ artificial muscles and enable faster dynamic actuation with reduced power consumption (enhanced efficiency), we coated graphene powder (electrical conductivity > 103 S/m) with mesoporous carbon and nickel nanoparticles.

“Mesostructured carbon” or “mesoporous carbon” refers to solid-based material, as defined by IUPAC [29]. These materials feature either ordered or disordered networks with pores distributed in the range of 2 to 50 nm, which can be broad or narrow. Mesoporous carbon offers excellent thermostability, high surface area, and a large pore volume, enhancing its functionality across various applications [30]. The presence of mesopores in carbon helps overcome limitations such as poor conductivity, structural integrity, and mass transport [31]. Mesoporous carbon materials find extensive applications in electrochemistry, energy storage, separation and adsorption, catalysis, and more. Sun et al. [32] demonstrated the use of metallic nanoparticles to enhance thermal conductivity by bridging two-dimensional (2D) materials. We utilized mesoporous carbon in the fabrication of conductive filaments [33,34]. Chen et al. [35] showcased the use of Ag/Ni metal mesh as a transparent conductive electrode for optoelectronic applications. Recently, Matharu et al. [15] demonstrated enhanced dynamic actuation and power consumption capability in mesoporous C-NiAg-PVA-coated mandrel-coiled TCP_FL_^NR^, showing the improvements with a nanomaterial-coated layer over a nylon 6,6 fishing line fiber.

In this study, nickel nanoparticles were incorporated into graphene powder and mesoporous carbon, with polyvinyl alcohol (PVA) was used as the binding material to improve thermal contacts, thereby enhancing the dynamic performance of TCP_FL_^NR^ [36]. The addition of graphene and removal of silver from the nanomaterial solution, makes this work distinct than our prior work in [15]. At approximately 80 °C, the interfaces between graphene powder and mesoporous carbon are connected by the nickel nanoparticles, ensuring improved thermal contacts. A one-pot magnetic stirrer process is employed to synthesize the graphene–mesoporous carbon–nickel–PVA solution, which is then coated onto the mandrel-coiled TCP_FL_^NR^ by manual shaking. The enhanced isotonic performance of the mandrel-coiled TCP_FL_^NR^ coated with graphene–mesoporous carbon–nickel–PVA (Graph-TCP) is investigated through cyclic operations with input power provided. 

The dynamic behavior of TCP_FL_^NR^ is dependent on its thermal properties. Materials like graphene have been used to improve the thermal conductivity of nylon fishing line since it has low thermal conductivity (0.230–0.380 W/m-K) [37]. To overcome the low thermal conductivity of nylon fishing line, high-quality monolayer graphene is used. Though the thermal conductivity of graphene is high, there is a thermal resistance between graphene particles due to weak interaction as the van der Waals force is prevalent. This restricts the improvement in the thermal properties of the graphene-based coating layer. Hence, metallic (Ni) nanoparticles are used to enhance thermal conductivity of the coating. Mesoporous carbon is utilized to improve the surface area of the coating and help cover the polymer surface. Since polyvinyl alcohol (PVA) has excellent binding strength and is commonly utilized as a binding agent for nanoparticles [38,39], it is used as the binding agent for this graphene–mesoporous C–Ni nanoparticle mixture on the surface of monofilament nylon 6 fishing line.

**Figure 1:**
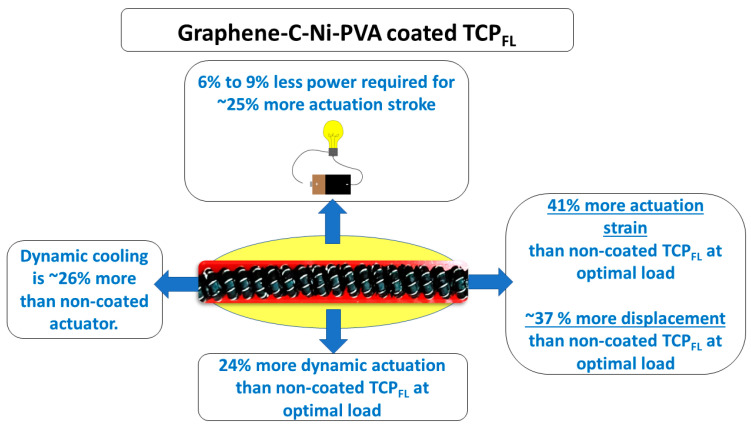
Overview of graphene–mesoporous C–Ni–PVA-coated mandrel-coiled TCP_FL_^NR^ artificial muscles. The maximum actuation strain at ~2 MPa load was 48% at 4 W power input and it could sustain 75,000 cyclic response at 0.1 Hz frequency.

The major contributions of this work (as illustrated in Figure 1) are as follows:

(i) Introduction of the novel graphene–mesoporous carbon–nickel–PVA-coated mandrel-coiled TCP_FL_^NR^, exhibiting approximately 41% higher actuation strain and about 37.5% greater actuation displacement compared to non-coated TCP_FL_^NR^ of similar length (60 mm unloaded and 69 mm loaded for 70 g pre-stress weight) under 0.27 A input current conditions.
(ii) Enhancement in dynamic actuation by approximately 24%, 36%, and 11% for pre-stress loads of 70 g, 100 g, and 150 g, respectively, with the novel nanomaterial-coated mandrel-coiled TCP_FL_^NR^, demonstrating the advantages of utilizing the graphene–mesoporous carbon–nickel–PVA solution.
(iii) Improvement in the cooling rate of the actuator by approximately 26%, 54%, and 11% for pre-stress loads of 70 g, 100 g, and 150 g, respectively, with the novel nanomaterial-coated mandrel-coiled TCP_FL_^NR^.
(iv) Reduction in power consumption ranging from approximately 6% to 9% for similar input current conditions (0.25 A, 0.27 A, 0.29 A), while providing an average of 25% more actuation strain.

## 2. Materials and Methods

### 2.1. Mandrel-Coiled TCP_FL_^NR^ Muscle Fabrication

**Figure 2:**
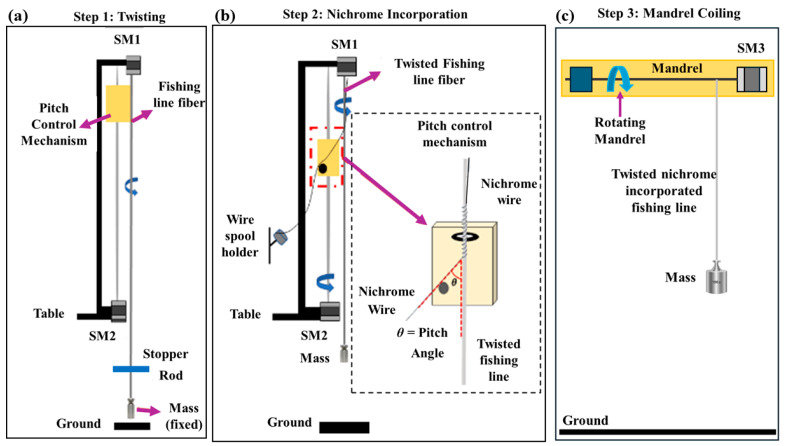
The mandrel-coiled TCP_FL_^NR^ actuator fabrication. (**a**) Twist insertion, (**b**) nichrome wire incorporation process, and (**c**) mandrel coiling process.

The fishing line, being a non-conductive material, requires the inclusion of nichrome heating wire to effectively heat the precursor material, facilitating convenient Joule heating for electrothermal actuation. The experimental setup, depicted in Figure 2a,b, is utilized to fabricate the mandrel-coiled TCP_FL_^NR^ muscles using a mandrel (1.4 mm diameter) for coiling. Nichrome wire (160 µm diameter) serves as the heating element for the monofilament 80 lb fishing line (0.8 mm diameter). Addressing the challenge of wrapping a thin nichrome wire conveniently, a custom fabrication setup, as shown in Figure 2, was employed. The various steps of the fabrication process are detailed below.

#### 2.1.1. Twist Insertion

A specific length of fishing line (1200 mm) was cut to accommodate the linear motion slider’s travel range. Both ends of the fishing line were secured with safety pins; one end was attached to the motor shaft (see Figure 2a). A deadweight of 500 g was affixed to the bottom end of the fishing line using a stopper shaft to introduce twist. The motor rotated at a speed of 300 rpm in a counterclockwise direction. After a few minutes, a twist was introduced into the fiber as it contracted in length, causing the 500 g load to gently ascend. Upon observing the initial coiling in the fiber, the motor was halted, and the twist insertion process was completed.

#### 2.1.2. Resistance Wire Incorporation

The next crucial step in the fabrication process involved incorporating the nichrome wire (as depicted in Figure 2b). Initially, the stopper utilized in the previous step was removed from the bottom end of the twist-inserted fishing line. A new untwisted fishing line with the 500 g weight attached to the bottom end of the twist-inserted fishing line was connected so that the weight hung just touching the ground and could rotate freely. The nichrome wire was affixed to the top end, and both the nichrome wire and the twist-inserted fishing line were positioned within the guide carriage and guide rod, as illustrated in Figure 2b. Both motors (Motor 1 and Motor 2) were activated at different speeds (Motor 1—300 rpm, Motor 2—150 rpm) until the nichrome incorporation was completed for the full length of the fishing line. At these speeds, the pitch of the nichrome-incorporated fiber was 0.42 mm. While the resistance wire incorporation was in progress, the wire was guided around the guide rod, and a 50 g weight was suspended to maintain tension during the wire incorporation process. This ensured a consistent pitch and wrapping angle.

#### 2.1.3. Mandrel Coiling Process

In Figure 2c, the mandrel coiling process for the nichrome-wrapped, twist-inserted fiber is depicted. A safety pin from the top end was attached to the stepper motor-3 (SM3) coupling, and the 500 g weight was replaced by a 200 g weight to facilitate easier coiling of the fiber by the stepper motor. A mandrel diameter of 1.4 mm was selected, and the stepper motor was initiated at a speed of 300 rpm in a counterclockwise direction. This allowed the fiber to coil and ensured that the finished product was homochiral in nature. Coiling the fiber in the opposite direction to its twist would have resulted in a heterochiral muscle [40].

#### 2.1.4. Thermal Annealing Process

The last stage of the mandrel-coiled TCP_FL_^NR^ muscle fabrication process involved muscle annealing, which was conducted in a furnace. The mandrel-coiled fiber, along with the mandrel rod, was placed inside the furnace preheated to a temperature of 180 °C. To maintain the muscle’s shape and pitch, both ends were secured to clamps on a metal plate, and the entire assembly was positioned inside the preheated furnace for 80 min. After 40 min, we inspected the muscle and inverted it to ensure uniform heating throughout.

### 2.2. Synthesis of Graphene–Mesoporous C–Ni–PVA Solution

**Figure 3:**
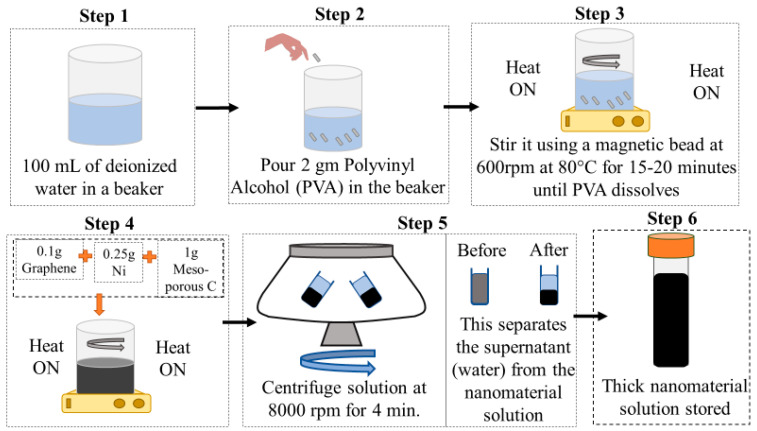
Synthesis of graphene-mesoporous C-Ni-PVA solution.

The nanomaterial solution preparation is clearly outlined in Figure 3. Firstly (Step 1), 100 mL of distilled water is measured into a 600 mL glass beaker. Then, 2 gm of PVA is added to the distilled water (Step 2), and the beaker is placed onto a magnetic stirrer with a magnetic bead at 800 rpm and 75–80 °C for approximately 15–20 min or until the PVA is fully dissolved in the water, resulting in a clear solution (Step 3). After this, 0.1 gm of graphene powder, 1 gm of mesoporous C, and 0.25 g of Ni nanoparticles are added to the solution, which is stirred at 600 rpm and 50 °C for about 3 h (Step 4). Once the solubility of the black solution is observed, the heat is turned off, and the reaction material is allowed to cool down while keeping the stirring at 600 rpm. The cooled-down solution is then centrifuged at 8000 rpm for approximately 4 to 5 min (Step 5), allowing excess water (supernatant) to separate from the thick solution. This supernatant is collected in a clean beaker, and some of it is added to the thick solution as needed for re-dissolving. This final synthesized solution is then used for coating the actuator.

### 2.3. Coating Process of Mandrel-Coiled TCP_FL_^NR^

**Figure 4:**
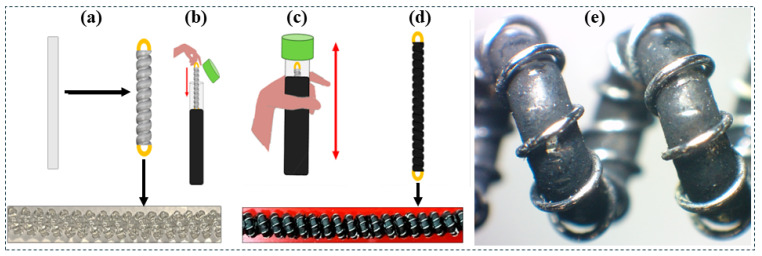
(**a**–**d**) Coating process of TCP_FL_^NR^ actuator. (**e**) Optical microscopy image of artificial muscle after completing lifecycle tests.

Once the mandrel-coiled TCP_FL_^NR^ is fabricated, as depicted in Figure 2, and the graphene-mesoporous C-Ni-PVA solution is synthesized, the coating process is initiated. The 60 mm long (3.4 mm diameter) non-coated TCP_FL_^NR^, as shown in Figure 4a, is immersed in the nanomaterial solution inside a vial (Figure 4b). To ensure thorough coating of the actuator, it is vigorously shaken manually for approximately 2 min (Figure 4c). After completion, the wet actuator is removed from the vial and placed in a preheated oven at 80 °C for 60 min to dry (Figure 4d). Once dried, the finished actuator is removed and crimped for use. Optical microscopy images (Figure 4e) were taken for the graphene-mesoporous C-Ni-PVA coated TCP_FL_^NR^. The image illustrates the consistency of the nanomaterial coating on the fishing line fiber. Note: This image was taken after the experiments were conducted.

### 2.4. Characterization of Coated TCP_FL_^NR^

**Figure 5:**
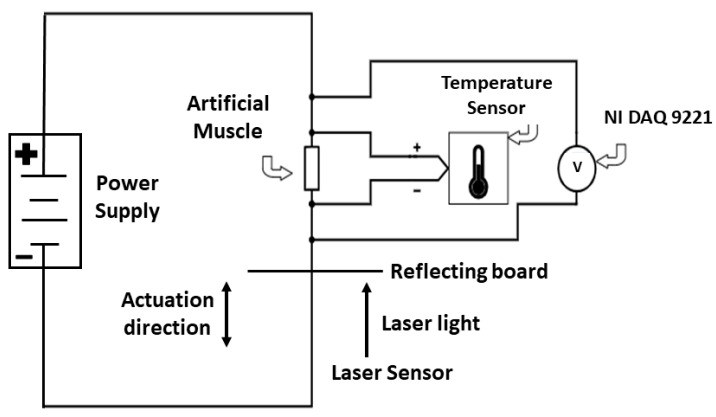
Characterization setup (schematic).

A characterization setup was employed to assess and compare the capabilities of both coated and non-coated TCP_FL_^NR^ muscles. This setup not only monitored the temperature increase in a muscle over time but also measured the voltage across the muscle when a constant current input was supplied through a BK Precision 9116 power source. As illustrated in Figure 5, the power supply delivered current to the muscle in series. A National Instruments DAQ 9221 module was connected in parallel with the muscle to measure the voltage across it, as voltage remains equivalent across parallel circuits. A thermistor was attached to the center of both actuators (coated and non-coated) to determine the temperature. A 70 g mass applied tension to the muscle at one end, while an opposing 70 g mass pulled on the other end. All these measurements were transmitted to a PC for data collection using LabView software. Similar characterization setups were utilized in previous studies on electrothermal artificial muscles [12,15].

## 3. Results and Discussion

The characterization setup outlined in Figure 5 was employed to conduct isotonic tests between the coated and non-coated TCP_FL_^NR^ artificial muscles. These tests aimed to ascertain the enhancement in performance following the coating of the TCP_FL_^NR^.

### 3.1. Characterization of Coated TCP_FL_^NR^

**Figure 6:**
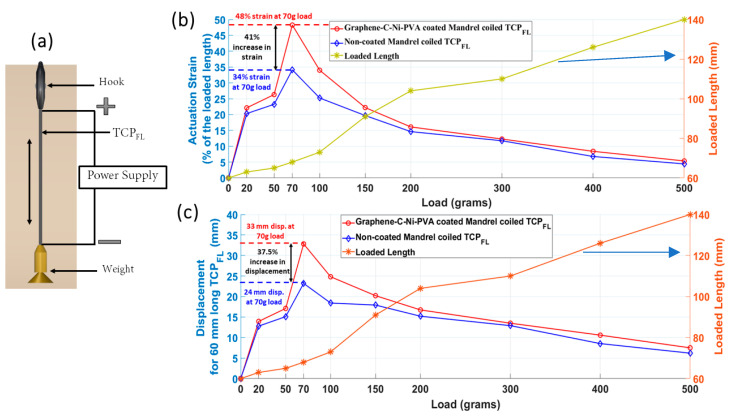
Characterization comparison of graphene-mesoporous C-Ni-PVA coated TCP_FL_^NR^ actuator and conventional TCP_FL_^NR^ actuator at 0.0285 Hz actuation frequency at different loads until 500 g. (**a**) Schematic diagram of training and characterization setup. (**b**) (Comparison of actuation strain with loaded length at different loads at similar input current for both actuators.) Comparison of actuation strain (% of loaded length) vs. different loads in grams of graphene-mesoporous C-Ni-PVA coated TCP_FL_^NR^ actuator (0.27 A, 15.8 V) and conventional TCP_FL_^NR^ actuator (0.27 A, 17.4 V) of 60 mm length each. (**c**) (Comparison of actuation displacement with loaded length at different loads at similar input current for both actuators.) Comparison of y-axis displacement with loaded length (mm) vs. different loads in grams of graphene-mesoporous C-Ni-PVA coated TCP_FL_^NR^ actuator (0.27 A, 15.8 V) and conventional TCP_FL_^NR^ actuator (0.27 A, 17.4 V) of 60 mm length each. The fishing line precursor fiber is 80 lb capacity and is 0.8 mm in diameter.

Displacement and actuation strain characterization were conducted by securing the nanomaterial-coated TCP_FL_^NR^ from a hook on one side and attaching various loads from the other side (refer to Figure 6a). Actuator characterization aimed to provide insights into their mechanical performance during a 15 s heating and 20 s cooling cycle (0.0285 Hz actuation frequency and approximately 43% duty cycle) under different loads up to 500 g. The blocking force, where the muscles break upon the provision of input current, was determined to be 500 g (4.9 N). This means, the blocking stress is 9.8 MPa (~10 MPa) when the force is normalized by the fishing line fiber area (0.8 mm diameter).

In Figure 6b, the load dependence of percentage tensile strain at the loaded length is illustrated, while Figure 6c depicts the same for actuation displacement of 60 mm long TCP_FL_^NR^ (both coated and non-coated) at a 0.0285 Hz (15 s heating, 20 s cooling) actuation frequency. The maximum displacement (~33 mm) for the graphene–mesoporous C–Ni–PVA-coated TCP_FL_^NR^ actuator occurred at a constant 70 g load, whereas the maximum displacement (~24 mm) for the conventional TCP_FL_^NR^ actuator of similar unloaded length (60 mm) occurred at a 70 g load. It is evident that the optimal range of actuation for these actuators lies between 20 g to 100 g.

The applied load is a crucial parameter as the tensile strain for the loaded length of the muscle depends on the applied load, given that the actuator undergoes significant elongation under increasing load. The maximum percentage of actuation strain for a constant load of 70 g for the graphene-mesoporous C-Ni-PVA coated TCP_FL_^NR^ actuator was noted to be approximately 48% at an input current of 0.27 A, resulting in 15.8 V and 4.4 W (time period of 35 s and a duty cycle of approximately 43%). This represented a 41% increase in actuation strain compared to the conventional TCP_FL_^NR^ actuator, with a difference of around 14% (0.27 A, 17.4 V, 4.7 W). It should be noted that both types of actuators were powered at similar input power capacities, beyond which they would quickly become damaged when the input power increases.

The loaded length of the TCP_FL_^NR^ actuator (Figure 6b) is directly proportional to the applied loads. Similarly, an improvement of approximately 37.5% can be observed for the coated actuator at 70 g (0.868 N or 1.4 MPa when normalized by the area) under similar input current conditions (0.27 A) for both the coated and non-coated actuators. 

### 3.2. Actuation Strain and Displacement Comparison with Time Variation of Coated and Non-Coated Actuator

**Figure 7:**
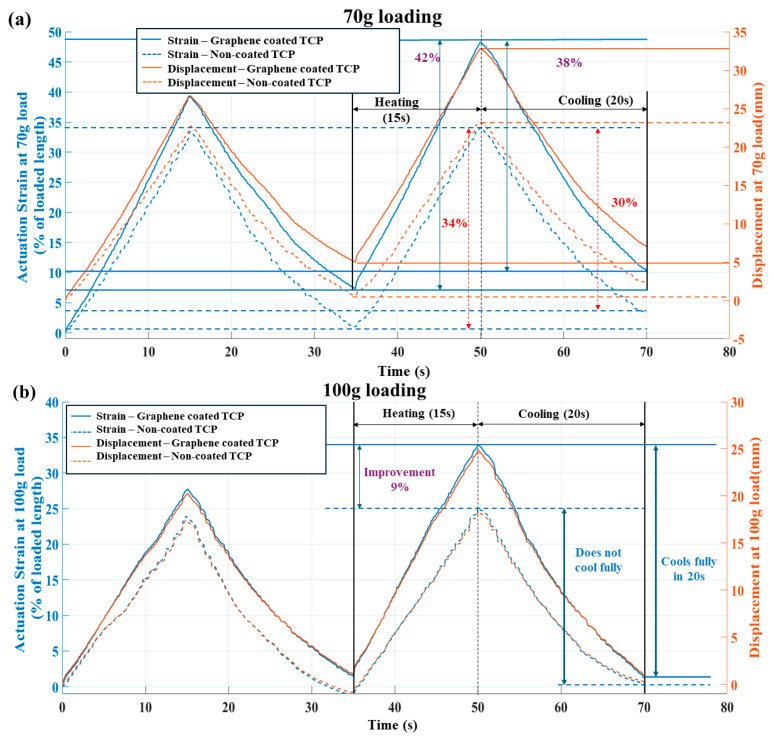

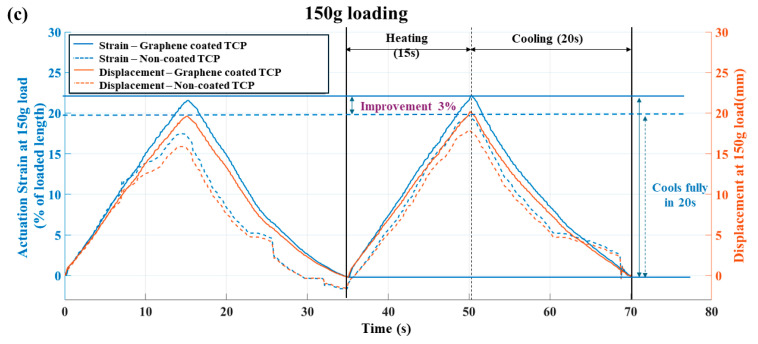
Dynamic actuation comparison of graphene-mesoporous C-Ni-PVA-coated TCP_FL_^NR^ actuator and conventional TCP_FL_^NR^ actuator at 0.0285 Hz actuation frequency at three different loads (70 g, 100 g, 150 g) for tensile actuation vs. time and y-axis displacement vs. time plots. (**a**) 70 g, (**b**) 100 g, (**c**) 150 g.

We also computed the actuation strain (% with loaded length) over time variation to establish a common metric for the same actuator of different lengths. Actuation strain serves as a metric to gauge the displacement performance for the same actuator with varying lengths. Figure 7a–c present the comparison of dynamic performance between both types of actuators, highlighting the faster heat dissipation of one over the other at different loadings. These graphs demonstrate the superior nature of the graphene-mesoporous C-Ni-PVA-coated TCP_FL_^NR^ actuator (powered at 4.3 W) compared to the conventional TCP_FL_^NR^ actuator (powered at 4.7 W). The three figures illustrate the tensile actuation with the y-axis representing displacement vs. time graphs at different loads (Figure 7a for 70 g load, Figure 7b for 100 g load, Figure 7c for 150 g load).

From the three graphs, it is evident that the dynamic response of the graphene-mesoporous C-Ni-PVA coated TCP_FL_^NR^ actuator is faster than that of the conventional TCP_FL_^NR^ actuator. It reaches the same tensile strain value as the conventional actuator approximately 4 to 6 s faster (approximately 40% faster than the total heating time of 15 s) for a 70 g load, while it is similar (5 to 6 s faster than the total heating time of 15 s) for a 100 g load. For a heavier weight (150 g), the coated actuator can achieve a similar actuation strain 2 to 3 s earlier (20% faster than the total heating time of 15 s).

For a similar input current of 0.27 A, the coated actuator exhibits approximately 24%, 36%, and 16% “more actuation strain”, respectively, than the non-coated actuator. Additionally, the coated actuator cools approximately 27%, 54%, and 11% “more” at 70 g, 100 g, and 150 g loading, respectively. It is also observed that the coated actuator returns fully to the original position for 100 g and 150 g loading within 20 s of cooling time; however, the same cannot be said for the non-coated conventional TCP_FL_^NR^.

### 3.3. Comparison of Power Consumed with Time for Coated and Non-Coated Actuator

**Figure 8:**
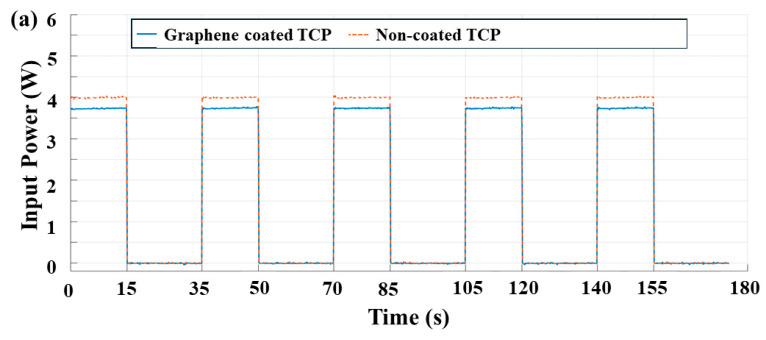

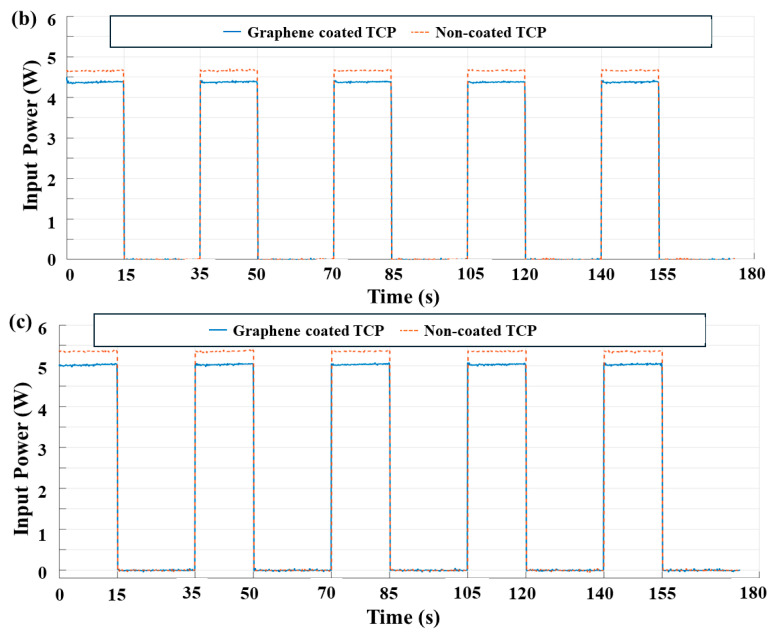
Comparison of power consumed (W) vs. time (s). Plots for coated and non-coated mandrel-coiled TCP_FL_^NR^ at different input currents, (**a**) 0.25 A, (**b**) 0.27 A, and (**c**) 0.29 A, at 0.0285 Hz (15 s on, 20 s off) frequency.

Figure 8a–c illustrate the improved power consumption based on the output voltages observed from the isotonic tests for constant input currents (0.25 A, 0.27 A, 0.29 A) for both coated and non-coated actuators. At 0.25 A input current, the voltage and power consumed for the coated TCP_FL_^NR^ were approximately 15 V (3.75 W), representing an improvement of about 6% over the non-coated TCP_FL_^NR^ (16 V, 4 W). Similarly, at 0.27 A input current, the output voltage/power consumed for the coated TCP_FL_^NR^ was approximately 15.9 V (4.3 W), which signifies an improvement of around 8.5% over the non-coated TCP_FL_^NR^ (~17.4 V, 4.7 W). At 0.29 A, we observed a ~7.4% improvement in power consumption of the coated TCP_FL_^NR^ (17.2 V, 5 W) compared to the non-coated TCP_FL_^NR^ (18.6 V, 5.4 W).

### 3.4. Comparison of Temperature Variation with Time for Coated and Non-Coated Actuator

**Figure 9:**
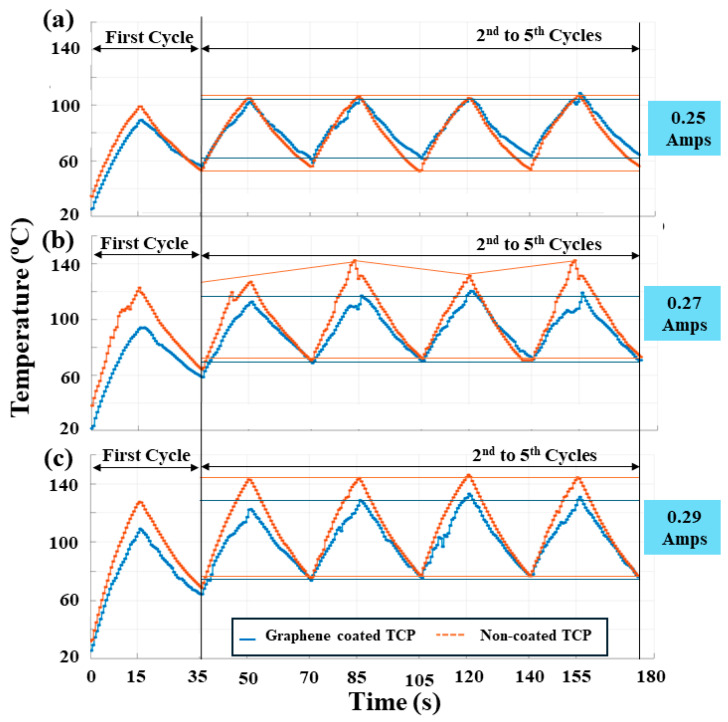
Comparison of temperature variation (°C) vs. time (s). Results for coated and non-coated mandrel-coiled TCP_FL_^NR^ at different input currents, (**a**) 0.25 A, (**b**) 0.27 A, and (**c**) 0.29 A, at 0.0285 Hz (15 s on, 20 s off) frequency.

Figure 9a–c depict the variation in temperature with time for input currents of 0.25 A, 0.27 A, and 0.29 A, with a heating time of 15 s and cooling time of 20 s (0.0285 Hz). The plots presented are for the first five cycles (first, second, third, fourth, and fifth cycles). It is evident that the starting temperature for cycles other than the first cycle is higher than room temperature. This occurs because after the first cycle, the actuator retains heat for both the muscles (coated and non-coated). However, it is noteworthy that the starting temperature of the second cycle is higher for the non-coated actuator (~57 °C for 0.25 A, ~65 °C for 0.27 A, and ~70 °C for 0.29 A) compared to the coated actuator (57 °C for 0.25 A, ~60 °C for 0.27 A, and ~65 °C for 0.29 A), indicating that the coated actuator cools faster with nanomaterial coating. The figures also demonstrate that the coated actuator rises to a lower temperature as the heat is distributed more uniformly throughout compared to a non-coated actuator.

### 3.5. Lifecycle Test of Graphene–Mesoporous C–Ni–PVA-Coated TCP_FL_^NR^

**Figure 10:**
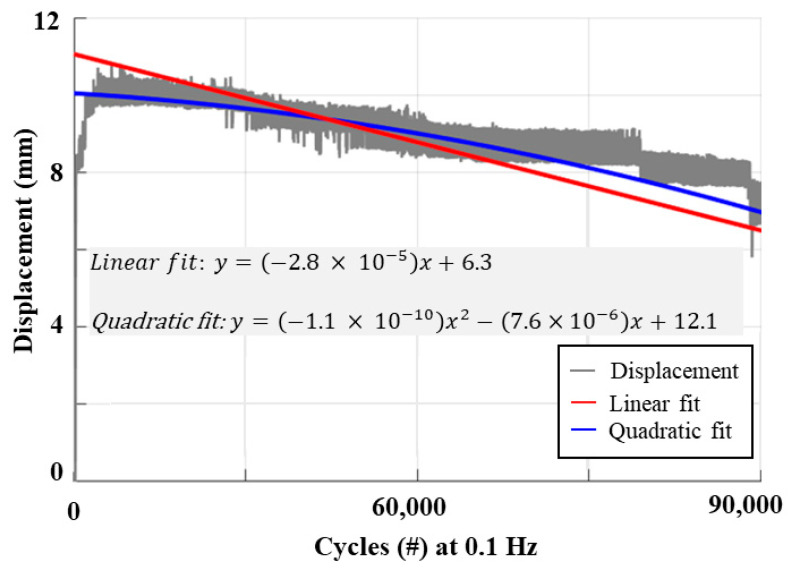
Tested lifecycle of graphene–mesoporous C–Ni–PVA-coated TCP_FL_^NR^ at 0.1 Hz and 0.27 A input current.

In the setup shown in Figure 5, we performed lifecycle/reliability tests of graphene–mesoporous C–Ni–PVA-coated TCP_FL_^NR^. Figure 10 illustrates the results of the lifecycle tests conducted for the 60 mm long actuator with a pre-stress load of 100 g and an input current of 0.27 A at 0.1 Hz (4s ON–6s OFF) actuation frequency. The actuator was tested for 90,000 actuation cycles at this frequency. It is seen from the results that the actuator shows uniform actuation and cooling until 75, 000 cycles, after which we start to see a gradual decline in performance. We also fitted the linear and quadratic curves to show the actuation performance trend. The lifecycle of this actuator is affected by varying frequency and applied input current, which can be investigated in future research.

## 4. Conclusions

In conclusion, the dynamic behavior, actuation strain/displacement, and power consumption of the mandrel-coiled TCP_FL_^NR^, a promising electrothermal artificial muscle, were significantly enhanced by surface treatment with graphene–mesoporous C–Ni–PVA coating. The coating solution was synthesized through a one-pot process using a magnetic stirrer and centrifuge. This solution was manually shaken and annealed to coat a dry, uniform layer of the material onto the fabricated actuator, which measured 3.4 mm in diameter and 60 mm in length. Graphene powder and Ni nanoparticles were combined with mesoporous carbon, using PVA as the binding agent. The coating improved the TCP_FL_^NR^’s thermal conductivity and actuation behavior.

The results of the characterization and comparative analysis between the coated and non-coated TCP_FL_^NR^ artificial muscles demonstrate the superior performance of the graphene–mesoporous C–Ni–PVA-coated TCP_FL_^NR^ actuator across multiple metrics. The coating significantly enhances both displacement and actuation strain, with the coated actuator achieving approximately 41% higher actuation strain at a constant 70 g load compared to the non-coated actuator. This improved performance is observed at similar input power levels, highlighting the efficiency of the coated actuator. The dynamic response analysis further underscores the advantages of the coated actuator, which consistently reaches target tensile strains faster than its non-coated counterpart. The coated actuator exhibits faster heat dissipation, achieving actuation strains 20–40% faster across various loads. Additionally, the coated actuator’s ability to return to its original position within the cooling timeframe at higher loads emphasizes its enhanced thermal management capabilities. Lifecycle tests were conducted for 75,000 actuation cycles at 0.1 Hz and it was seen that the actuator can actuate for a greater number of cycles. Table 1 shows the comparison between graphene–C–Ni–PVA-coated TCP_FL_^NR^ and other comparable electrothermal actuators.

**Figure 11:**
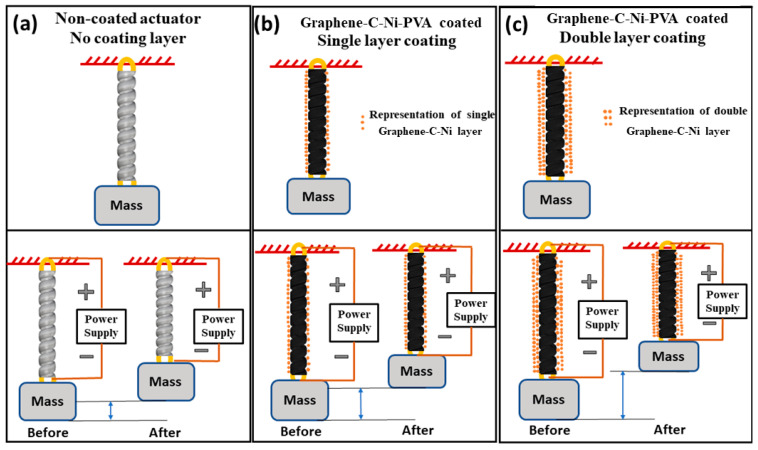
Proposed work showing the difference in performance of the TCP fishing line actuator; (**a**) non-coated, (**b**) single-layer graphene–mesoporous C–Ni–PVA-coated, (**c**) double-layer graphene–mesoporous C–Ni–PVA-coated.

Power consumption studies reveal a marked improvement for the coated TCP_FL_^NR^ actuator, with reductions in power consumption ranging from 6% to 8.5% across different input currents. This indicates not only improved mechanical performance but also a reduction in energy requirements, making the coated actuator more sustainable for prolonged use. A temperature variation analysis supports the earlier findings, showing that the coated actuator achieves a more uniform temperature distribution and faster cooling rates. This thermal advantage contributes to its overall superior performance and durability, as the actuator is less prone to thermal damage. In conclusion, the incorporation of graphene–mesoporous C–Ni–PVA coating in TCP_FL_^NR^ actuators significantly enhances their mechanical and thermal performance. These findings suggest that such coatings could be pivotal in the development of more efficient and durable artificial muscles, with broad applications in robotics and biomedical devices. Future work should explore the long-term stability and potential scalability of this coating technology to fully realize its practical benefits. In the future, we will focus on enhancing the performance of artificial muscles by adjusting the volume ratios of various materials in the nanomaterial coating. We will characterize these enhancements based on input current, actuation displacement, actuation frequency, and actuator lifecycle. Underwater applications like the actuation of soft robotic jellyfish with similar nanomaterial-coated TCP_FL_^NR^ actuators have been seen in the literature [16]. We can utilize the graphene–mesoporous C–Ni–PVA-coated TCP_FL_^NR^ in similar applications for actuating underwater soft robots. In the future, we would characterize the graphene–mesoporous C–Ni–PVA-coated TCP_FL_^NR^ by applying the coating a different number of times as shown in Figure 11a–c.

## Figures and Tables

**Table 1:** Comparison between graphene–C–Ni–PVA-coated TCP_FL_^NR^ and other comparable electrothermal actuators. Dash entries indicate data not found.

Actuation Technologies	This Work	SMA Wire [41]	TCP-6ply [12]	TCP Fishing Line with Heater [13,42]	Fishing Line Hydrothermal Mandrel-Coiled [43]	Coiled SMA [41]	**TCP Mandrel-Coiled Muscle** [36]
**Material**	Graphene–C–Ni–PVA-coated TCP_FL_^NR^	Mandrel-coiled nylon 6, fishing line with 0.16 mm nichrome	Silver-coated nylon 66	Nylon 66, fishing line with 0.08 mm nichrome	Nylon 66, fishing line	Nickel and titanium (NiTi)	Mandrel-coiled nylon 6, fishing line with 0.08 mm nichrome
**Actuation type**	Electrothermal	Electrothermal	Electrothermal	Electrothermal	Hydrothermal	Electrothermal	Electrothermal
**Precursor fiber** **diameter (mm)**	0.8	0.127	0.2	0.8	0.8	0.381	0.8
**Precursor filament type**	Monofilament	N/A	Multifilament	Monofilament	Monofilament	N/A	Monofilament
**Outer diameter (mm)**	3.4	N/A	2.4	2.8	3.3	0.10	4.5
**Mandrel usage #**	Yes	No	No	No	Yes	Yes	Yes
**Resistance (Ω/mm)**	1	1.9 (Ω/inch)	0.031	2	-----------	0.00827	2.5
**Force lifting capability at max strain (MPa)**	~2	~4.4	~1.96	~1.56	300 g	0.3	1.69
**Actuation stroke**	48% ^†^	4–8%	17–20%	38%	30% at 90 °C water	120% at 0.25 Hz	53% *
**Lifecycle (cycles)**	Tested until 90,000 cycles	~10^6^ cycles in air	787 in water at 0.25 Hz, 25% duty cycle	2400 in air at 0.009 Hz and 1% duty cycle	-----------	~10^4^ cycles in water	Tested until 2300 cycles

^†^ Maximum actuation strain. * Diameter used is more than conventional TCP fabricated by us. # Mandrel diameter size can differ, changing output characteristics of artificial muscle in terms of strain (%), load-carrying capacity, displacement, etc.

## Data Availability

The original contributions presented in the study are included in the article, further inquiries can be directed to the corresponding authors.

## References

[1] Majidi C. (2019). Soft-matter engineering for soft robotics. Adv. Mater. Technol..

[2] Rus D., Tolley M.T. (2015). Design, fabrication and control of soft robots. Nature.

[3] Jeong J., Hyeon K., Jang S.-Y., Chung C., Hussain S., Ahn S.-Y., Bok S.-K., Kyung K.-U. (2022). Soft wearable robot with shape memory alloy (SMA)-based artificial muscle for assisting with elbow flexion and forearm supination/pronation. IEEE Robot. Autom. Lett..

[4] Matharu P.S., Wang Z., Costello J.H., Colin S.P., Baughman R.H., Tadesse Y.T. (2023). SoJel—A 3D printed jellyfish-like robot using soft materials for underwater applications. Ocean Eng..

[5] Almubarak Y., Punnoose M., Maly N.X., Hamidi A., Tadesse Y. (2020). KryptoJelly: A jellyfish robot with confined, adjustable pre-stress, and easily replaceable shape memory alloy NiTi actuators. Smart Mater. Struct..

[6] Higueras-Ruiz D., Shafer M., Feigenbaum H. (2021). Cavatappi artificial muscles from drawing, twisting and coiling polymer tubes. Sci. Robot..

[7] Higueras-Ruiz D.R., Feigenbaum H.P., Shafer M.W. (2022). Material-based modeling of cavatappi artificial muscles. Smart Mater. Struct..

[8] Joshi A., Kulkarni A., Tadesse Y. (2019). FludoJelly: Experimental study on jellyfish-like soft robot enabled by soft pneumatic composite (SPC). Robotics.

[9] Xavier M.S., Tawk C.D., Zolfagharian A., Pinskier J., Howard D., Young T., Lai J., Harrison S.M., Yong Y.K., Bodaghi M. (2022). Soft Pneumatic Actuators: A Review of Design, Fabrication, Modeling, Sensing, Control and Applications. IEEE Access.

[10] Mao G., Drack M., Karami-Mosammam M., Wirthl D., Stockinger T., Schwödiauer R., Kaltenbrunner M. (2020). Soft electromagnetic actuators. Sci. Adv..

[11] Haines C.S., Lima M.D., Li N., Spinks G.M., Foroughi J., Madden J.D.W., Kim S.H., Fang S., de Andrade M.J., Göktepe F. (2014). Artificial Muscles from Fishing Line and Sewing Thread. Science.

[12] Hamidi A., Almubarak Y., Rupawat Y., Warren J., Tadesse Y. (2020). Poly-saora robotic jellyfish: Swimming underwater by twisted and coiled polymer actuators. Smart Mater. Struct..

[13] Matharu P.S., Ghadge A.A., Almubarak Y., Tadesse Y. (2022). Jelly-Z: Twisted and coiled polymer muscle actuated jellyfish robot for environmental monitoring. ACTA IMEKO.

[14] Matharu P.S., Gong P., Guntaka K.P.R., Almubarak Y., Jin Y., Tadesse Y.T. (2023). Jelly-Z: Swimming performance and analysis of twisted and coiled polymer (TCP) actuated jellyfish soft robot. Sci. Rep..

[15] Matharu P.S., Song Y., Gandhi U., Tadesse Y. (2023). Fabrication and Characterization of Mesoporous Carbon-Nickel Silver Powder-Poly (Vinyl Alcohol) Coated Mandrel-Coiled TCPFL Artificial Muscles for Enhanced Performance. Smart Materials, Adaptive Structures and Intelligent Systems.

[16] Matharu P.S., Ovy S.M.A.I., Singh A.P., Song Y., Gandhi U., Tadesse Y. Jelly-Z 2.0: 3D Printed Soft Jellyfish Robot Actuated With Self-Coiled CNT-C-Ni-PVA Coated TCPFL. Proceedings of the ASME 2023 Conference on Smart Materials, Adaptive Structures and Intelligent Systems.

[17] Lee J.A., Li N., Haines C.S., Kim K.J., Lepró X., Ovalle-Robles R., Kim S.J., Baughman R.H. (2017). Electrochemically Powered, Energy-Conserving Carbon Nanotube Artificial Muscles. Adv. Mater..

[18] Di J., Fang S., Moura F.A., Galvão D.S., Bykova J., Aliev A., de Andrade M.J., Lepró X., Li N., Haines C. (2016). Strong, Twist-Stable Carbon Nanotube Yarns and Muscles by Tension Annealing at Extreme Temperatures. Adv. Mater..

[19] Lima M.D., Li N., de Andrade M.J., Fang S., Oh J., Spinks G.M., Kozlov M.E., Haines C.S., Suh D., Foroughi J. (2012). Electrically, Chemically, and Photonically Powered Torsional and Tensile Actuation of Hybrid Carbon Nanotube Yarn Muscles. Science.

[20] Piao C., Jang H., Lim T., Kim H., Choi H.R., Hao Y., Suk J.W. (2019). Enhanced dynamic performance of twisted and coiled soft actuators using graphene coating. Compos. Part B Eng..

[21] Piao C., Suk J.W. (2020). Graphene/silver nanoflower hybrid coating for improved cycle performance of thermally-operated soft actuators. Sci. Rep..

[22] Pop E., Varshney V., Roy A.K. (2012). Thermal properties of graphene: Fundamentals and applications. MRS Bull..

[23] Su Y., Li J.J., Weng G.J. (2018). Theory of thermal conductivity of graphene-polymer nanocomposites with interfacial Kapitza resistance and graphene-graphene contact resistance. Carbon.

[24] Fu Y., Hansson J., Liu Y., Chen S., Zehri A., Samani M.K., Wang N., Ni Y., Zhang Y., Zhang Z.-B. (2020). Graphene related materials for thermal management. 2D Mater..

[25] Sun Y., Chen L., Cui L., Zhang Y., Du X. (2018). Molecular dynamics simulation of the effect of oxygen-containing functional groups on the thermal conductivity of reduced graphene oxide. Comput. Mater. Sci..

[26] Yeom Y.S., Cho K.Y., Seo H.Y., Lee J.S., Im D.H., Nam C.Y., Yoon H.G. (2020). Unprecedentedly high thermal conductivity of carbon/epoxy composites derived from parameter optimization studies. Compos. Sci. Technol..

[27] Balandin A.A., Ghosh S., Bao W., Calizo I., Teweldebrhan D., Miao F., Lau C.N. (2008). Superior Thermal Conductivity of Single-Layer Graphene. Nano Lett..

[28] Chen S., Moore A.L., Cai W., Suk J.W., An J., Mishra C., Amos C., Magnuson C.W., Kang J., Shi L. (2011). Raman Measurements of Thermal Transport in Suspended Monolayer Graphene of Variable Sizes in Vacuum and Gaseous Environments. ACS Nano.

[29] Rouquerol J., Avnir D., Fairbridge C.W., Everett D.H., Haynes J.M., Pernicone N., Ramsay J.D.F., Sing K.S.W., Unger K.K. (1994). Recommendations for the characterization of porous solids (Technical Report). Pure Appl. Chem..

[30] Rahman M.M., Ara M.G., Alim M.A., Uddin M.S., Najda A., Albadrani G.M., Sayed A.A., Mousa S.A., Abdel-Daim M.M. (2021). Mesoporous Carbon: A Versatile Material for Scientific Applications. Int. J. Mol. Sci..

[31] Liu B., Liu L., Yu Y., Zhang Y., Chen A. (2020). Synthesis of mesoporous carbon with tunable pore size for supercapacitors. New J. Chem..

[32] Sun J., Yao Y., Zeng X., Pan G., Hu J., Huang Y., Sun R., Xu J.-B., Wong C.-P. (2017). Preparation of boron nitride nanosheet/nanofibrillated cellulose nanocomposites with ultrahigh thermal conductivity via engineering interfacial thermal resistance. Adv. Mater. Interfaces.

[33] Jain S.K., Tadesse Y. (2019). Fabrication of polylactide/carbon nanopowder filament using melt extrusion and filament characterization for 3D printing. Int. J. Nanosci..

[34] Potnuru A., Tadesse Y. (2019). Investigation of polylactide and carbon nanocomposite filament for 3D printing. Prog. Addit. Manuf..

[35] Chen X., Guo W., Xie L., Wei C., Zhuang J., Su W., Cui Z. (2017). Embedded Ag/Ni Metal-Mesh with Low Surface Roughness As Transparent Conductive Electrode for Optoelectronic Applications. ACS Appl. Mater. Interfaces.

[36] Wu L., Chauhan I., Tadesse Y. (2018). A novel soft actuator for the musculoskeletal system. Adv. Mater. Technol..

[37] MatWeb Overview of Materials for Nylon 6, Cast. https://matweb.com/search/DataSheet.aspx?MatGUID=8d78f3cfcb6f49d595896ce6ce6a2ef1&ckck=1.

[38] Wu Y., Chen E., Weng X., He Z., Chang G., Pan X., Liu J., Huang K., Huang K., Lei M. (2022). Conductive Polyvinyl Alcohol/Silver Nanoparticles Hydrogel Sensor with Large Draw Ratio, High Sensitivity and High Stability for Human Behavior Monitoring. Eng. Sci..

[39] Elango J., Zamora-Ledezma C., Alexis F., Wu W., de Val J.E.M.-S. (2023). Protein Adsorption, Calcium-Binding Ability, and Biocompatibility of Silver Nanoparticle-Loaded Polyvinyl Alcohol (PVA) Hydrogels Using Bone Marrow-Derived Mesenchymal Stem Cells. Pharmaceutics.

[40] Cherubini A., Moretti G., Vertechy R., Fontana M. (2015). Experimental characterization of thermally-activated artificial muscles based on coiled nylon fishing lines. AIP Adv..

[41] Dynalloy (2021). FLEXINOL® Actuator Spring Technical and Design Data.

[42] Hamidi A., Almubarak Y., Tadesse Y. (2019). Multidirectional 3D-printed functionally graded modular joint actuated by TCPFL muscles for soft robots. Bio-Des. Manuf..

[43] Wu L., de Andrade M.J., Rome R.S., Haines C., Lima M.D., Baughman R.H., Tadesse Y. (2015). Nylon-muscle-actuated robotic finger. Proc. SPIE.

